# Breast metastasis of cervical cancer: A case report and systematic literature review

**DOI:** 10.3389/fonc.2022.974592

**Published:** 2022-09-14

**Authors:** Zhang Jian-yong, Zeng Guang-ping, Wang Xue, Zhang Shi-min, Zhao Zhen-guo

**Affiliations:** Department of Oncology, First People’s Hospital of Guangyuan, Guangyuan, China

**Keywords:** cervical cancer, breast metastases, interstitial brachytherapy, immunohistochemical, systemic chemotherapy

## Abstract

It has been reported that extramammary malignant tumors metastasize to the breast, but cervical cancer metastasis to the breast is very rare. At present, there are only dozens of reports about cervical cancer metastasis to breast in the world. It is difficult to distinguish between primary breast cancer and metastatic breast cancer. We report a 44-year-old woman who underwent surgery, chemotherapy, and radiotherapy for cervical cancer 5 years ago. Then, she was hospitalized for finding a left breast mass measured 2.9 × 2.7 cm in chest CT. Pathological examination combined with immunohistochemical staining showed that the mass came from the cervix. Then, the patient received systematic chemotherapy and interstitial brachytherapy (IB) for the breast mass and got a great result. Cervical cancer rarely metastasizes to the breast. In this case, we confirmed the diagnosis of breast mass by histopathological examination and immunohistochemistry. IB achieved a good result in the treatment of the breast mass. We hope to provide reference of prognosis and treatment when facing this situation by presenting this case.

## Introduction

Cervical cancer is one of the most common malignant tumors of female reproductive tract. It ranks fourth among the cancers in women (1). Breast metastasis of cervical cancer is very rare, so most clinicians lack experience in the diagnosis and treatment of this kind of patients. According to post-mortem autopsy reports, the incidence rate of non-mammary malignancies metastasizing to the breast is about 1.7%–6.6% (2). Clinical reports estimated that the incidence is lower, about 0.4%–3% (3).

The treatment methods and prognosis of metastatic breast cancer (MBC) are different from primary breast cancer (PBC). Therefore, it is very important to distinguish MPC from PBC for patients with breast tumors to receive correct and rapid treatment. In this case report, we describe a patient with cervical cancer who underwent surgery and radiotherapy 5 years ago. She presented with breast mass 2 years ago; on further examination, she was diagnosed as squamous cell carcinoma of cervical cancer with metastasis the breast. Therapeutic efficacy was very good with the treatment of interstitial brachytherapy IB and systemic chemotherapy.

## Case report

A 44-year-old woman presented with irregular vaginal bleeding for more than 1 year. The needle biopsy result revealed poorly differentiated squamous cell carcinoma. The patient received total hysterectomy with bilateral salpingo-oophorectomy and pelvic lymph node dissection after two cycles of chemotherapy (paclitaxel + cisplatin). Postoperative pathology showed that cervical invasive poorly differentiated squamous cell carcinoma, and eight of the 14 dissected lymph nodes were involved. Then, concurrent chemoradiotherapy and three-dimensional brachytherapy were given after surgery. No abnormality was found in the follow-up of gynecological outpatient department.

**Figure 1:**
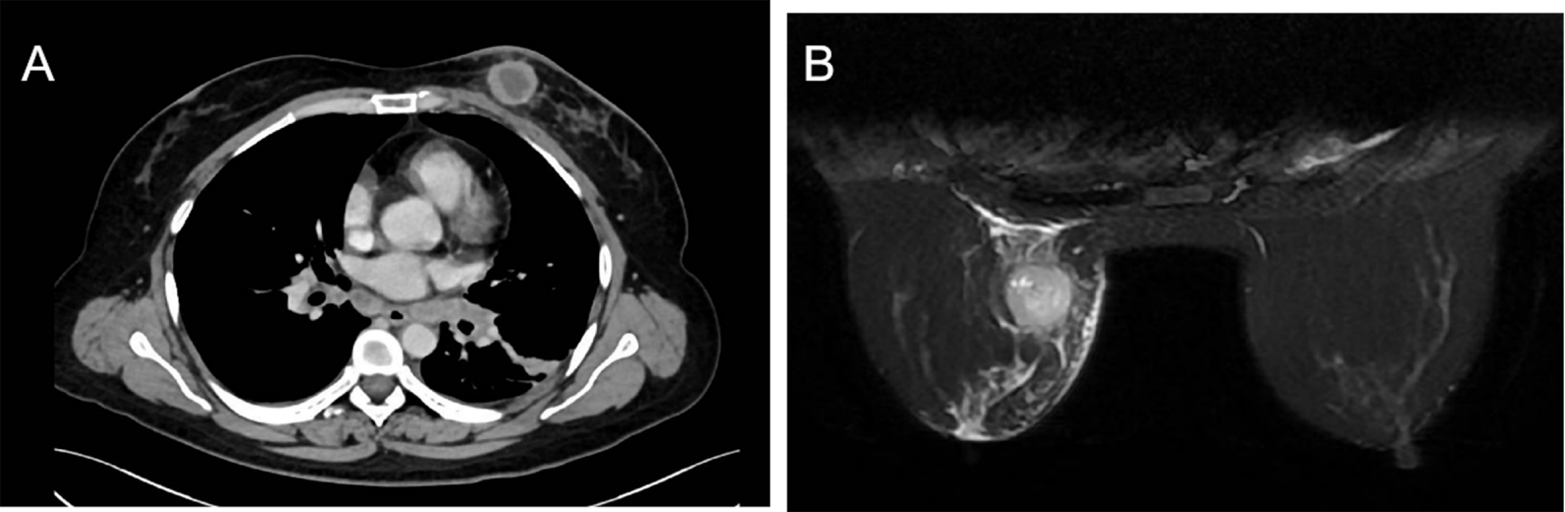
Chest CT. Chest CT before treatment **(A)**. Chest MRI before treatment **(B)**.

**Figure 2:**
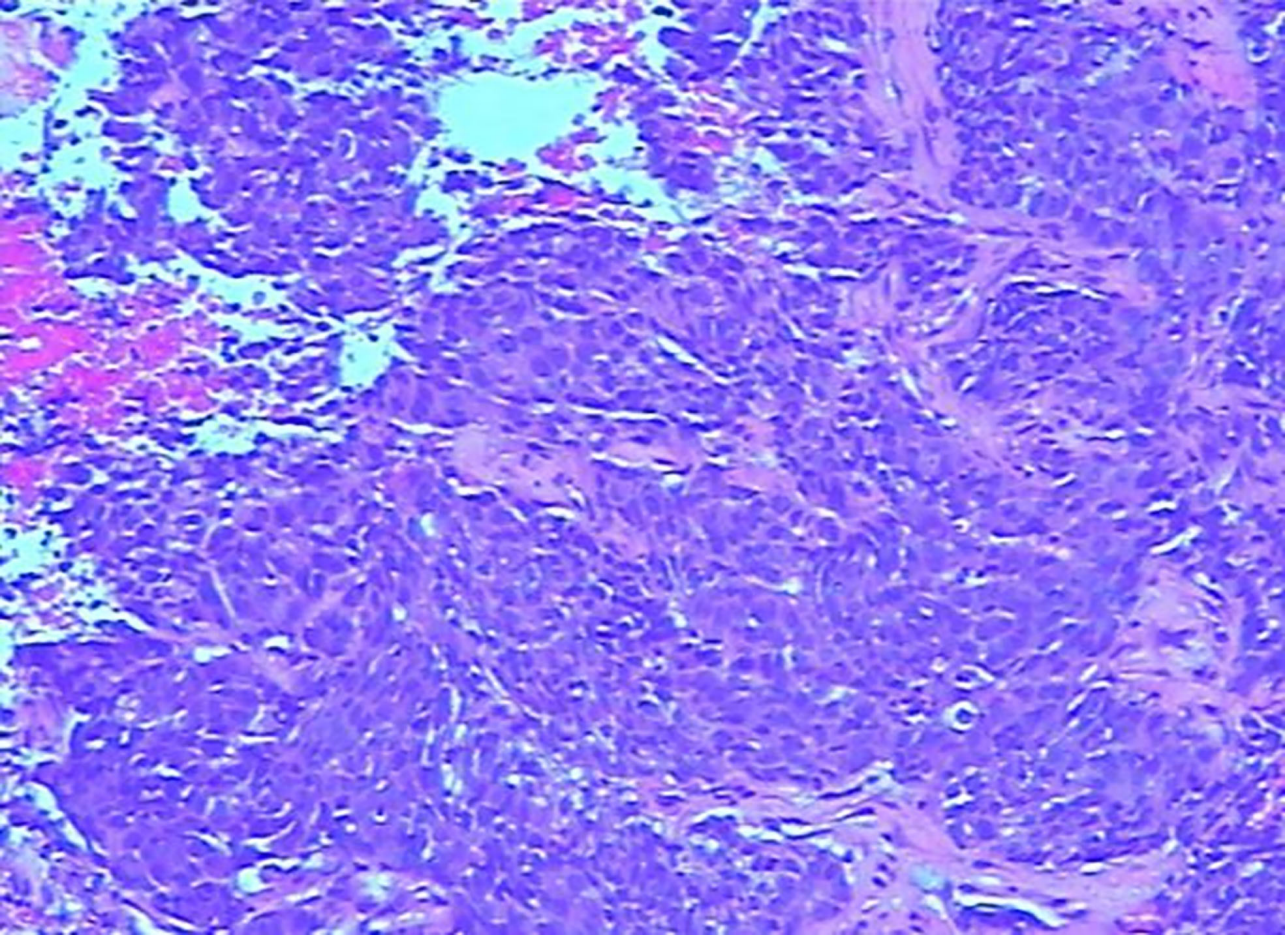
Puncture pathology of left breast mass.

**Figure 3:**
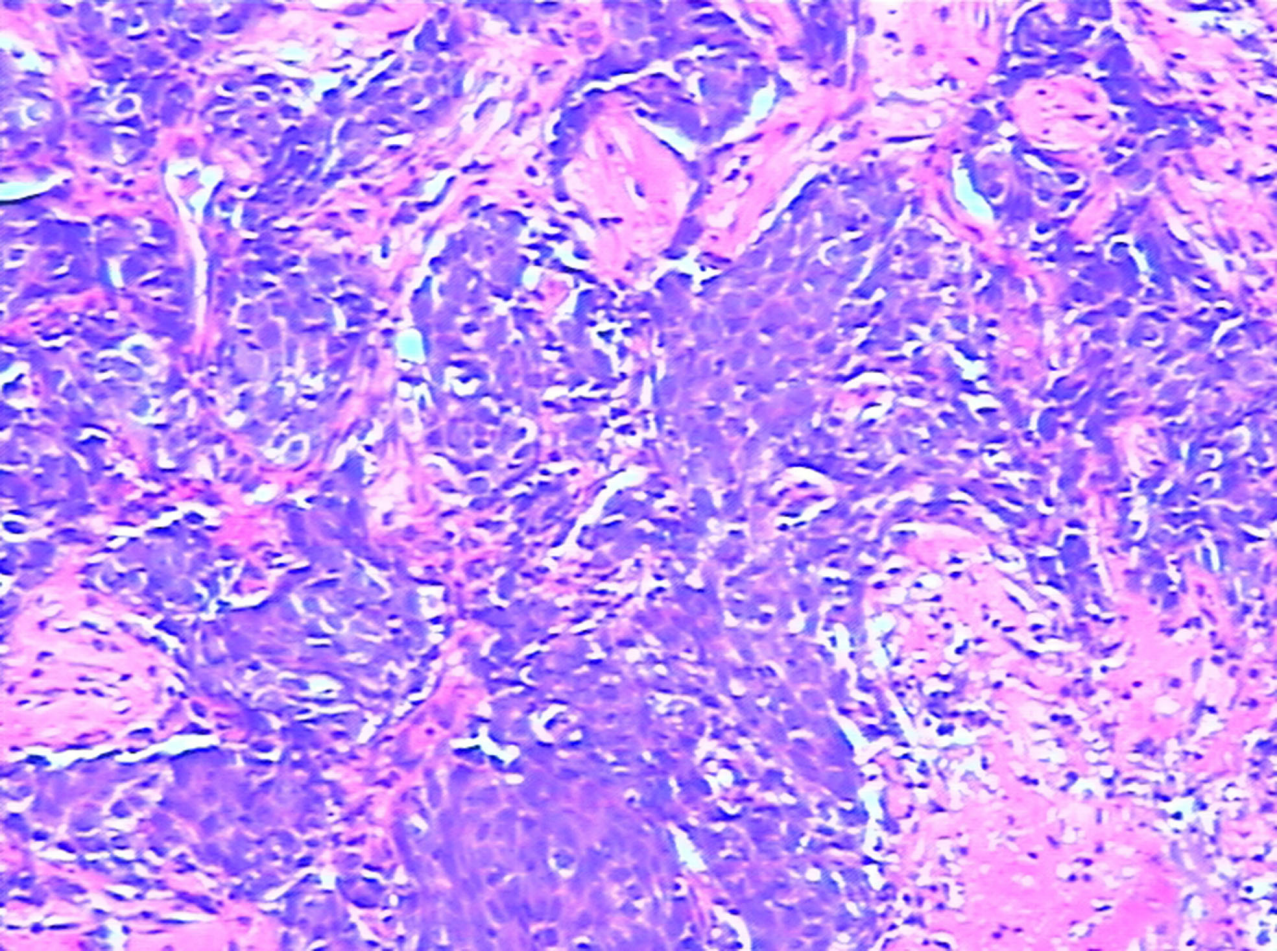
Puncture pathology of the left porta of the lung.

Three years after surgery, the patient was admitted to the hospital with a lump in her left breast. Physical examination: A mass with a size of about 3 cm was found in the inner and lower quadrant of the left breast. It was hard, no pain, no swelling, no ulcer, good mobility, and no orange peel–like change. No obvious mass was found in the right breast and double armpits. Chest CT and MRI showed a size of 2.9 × 2.7-cm space occupying lesion in the left breast, mediastinum, and left hilar lymph node metastasis (**Figures 1A, B**). Gynecological examination and pelvic MRI showed no abnormalities. Metastatic squamous cell carcinoma from cervix was considered in the pathological biopsy of left breast mass (**Figure 2**) and left hilar (**Figure 3**), and the immunohistochemical examination of left breast tumor cells showed ER(−), PR(−), HER-2(2+), CK5/6(+), P63(+), SMMHC(−), GTA-3(+), EGFR(+), P53(wild type), Ki67(80%+), and P16(+).

**Figure 4:**
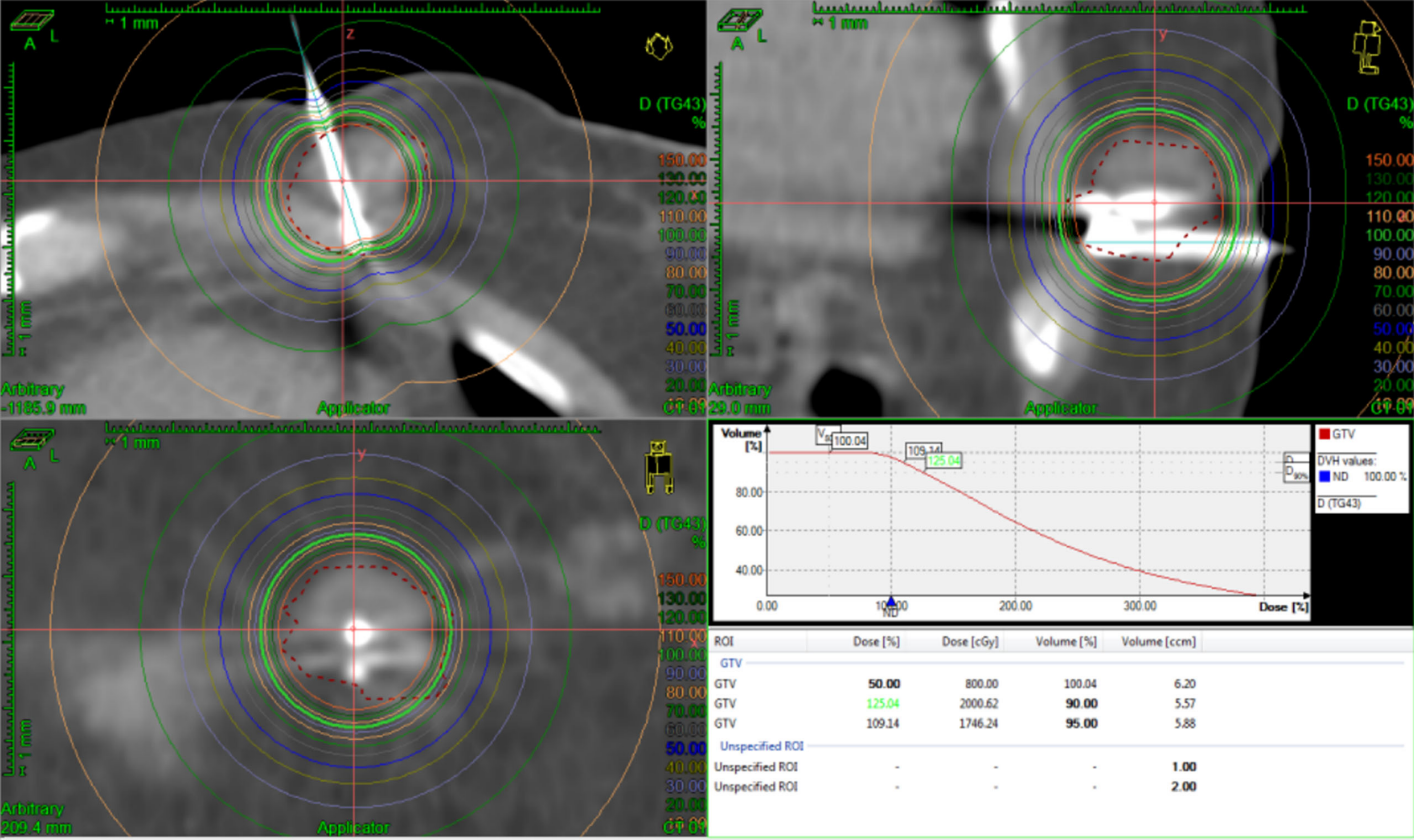
Three-dimensional dose distribution and DVH of the first fraction of IB.

**Figure 5:**
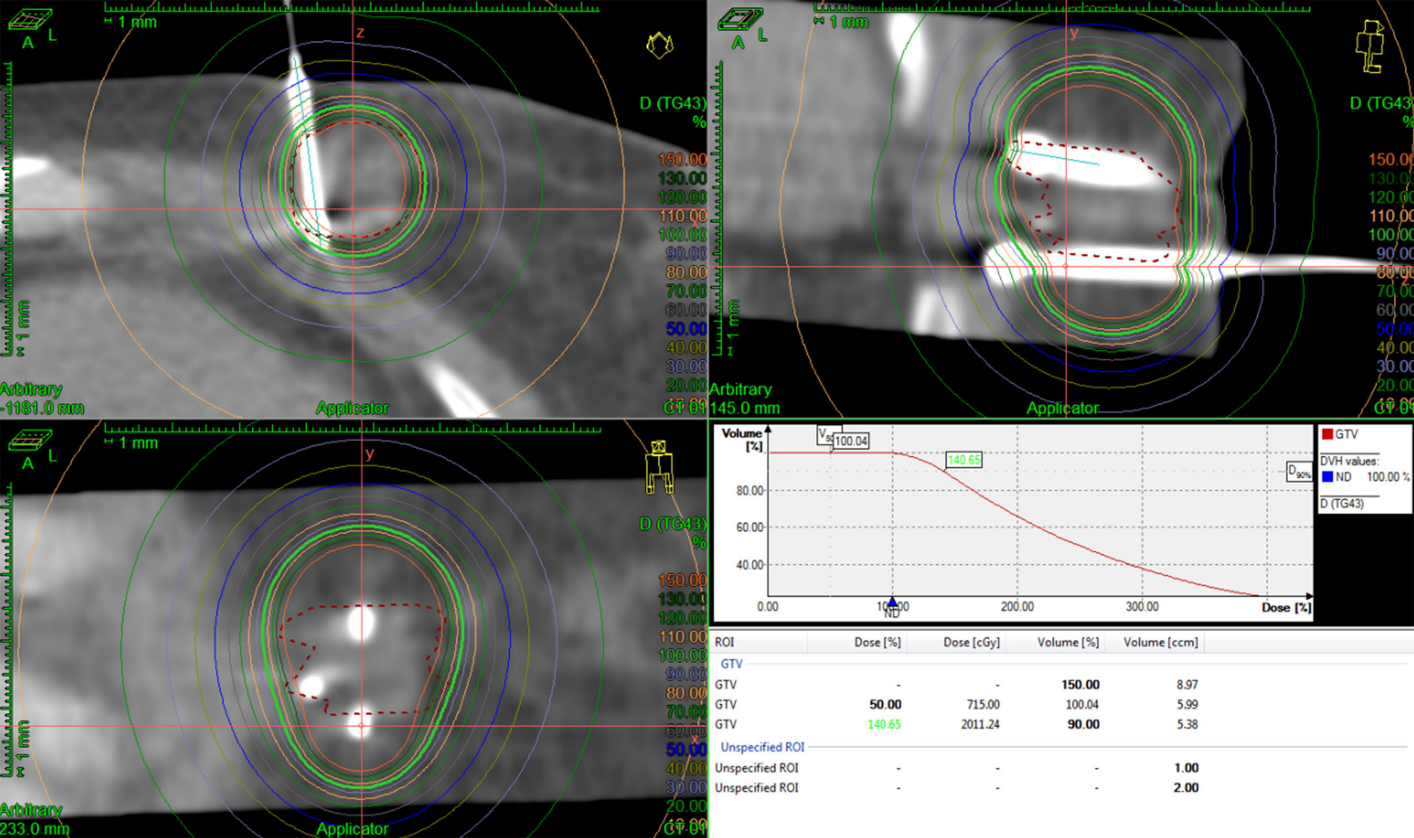
Three-dimensional dose distribution and DVH of the second fraction of IB.

**Figure 6:**
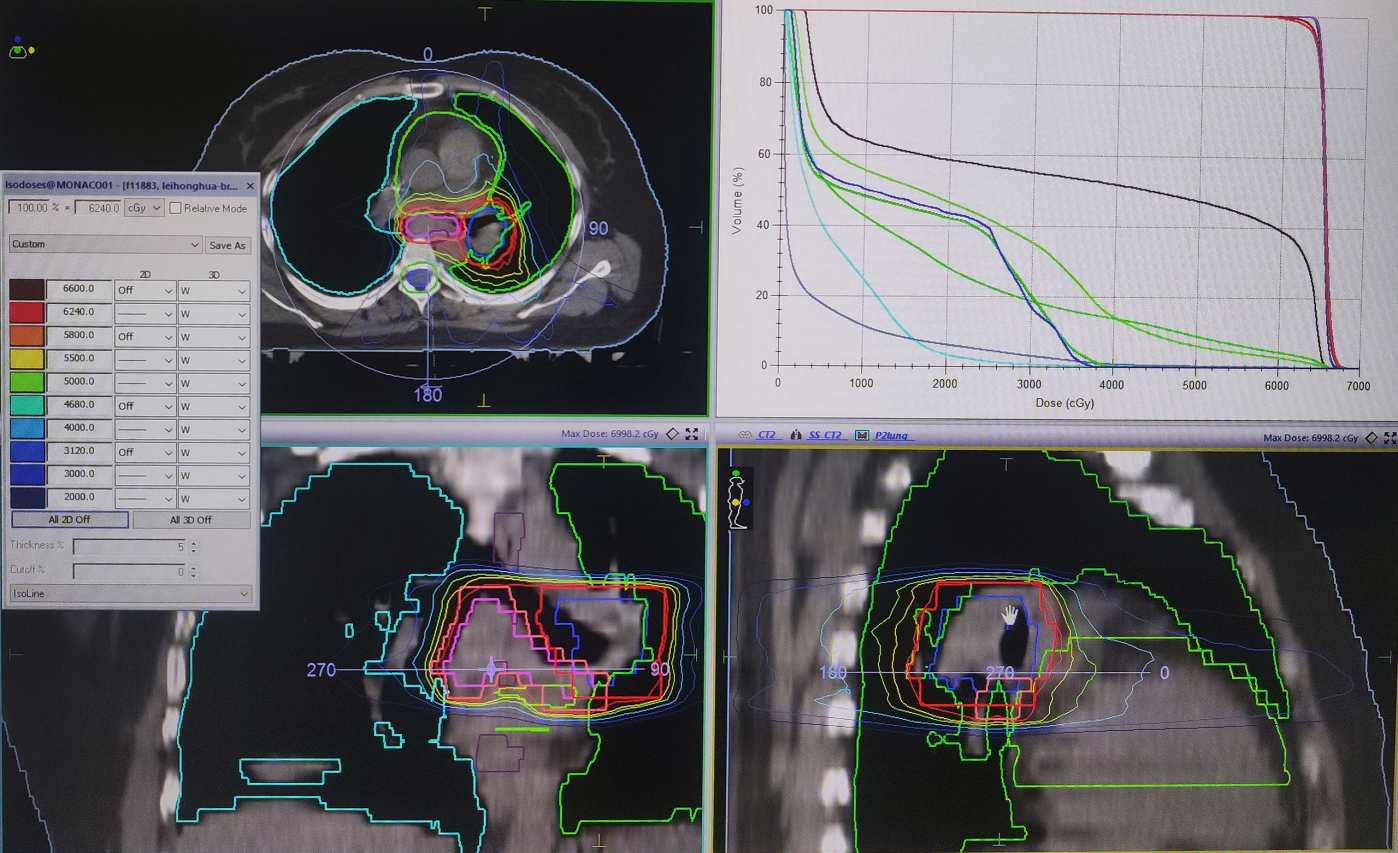
Three-dimensional dose distribution and DVH of the left porta of the lung.

**Figure 7:**
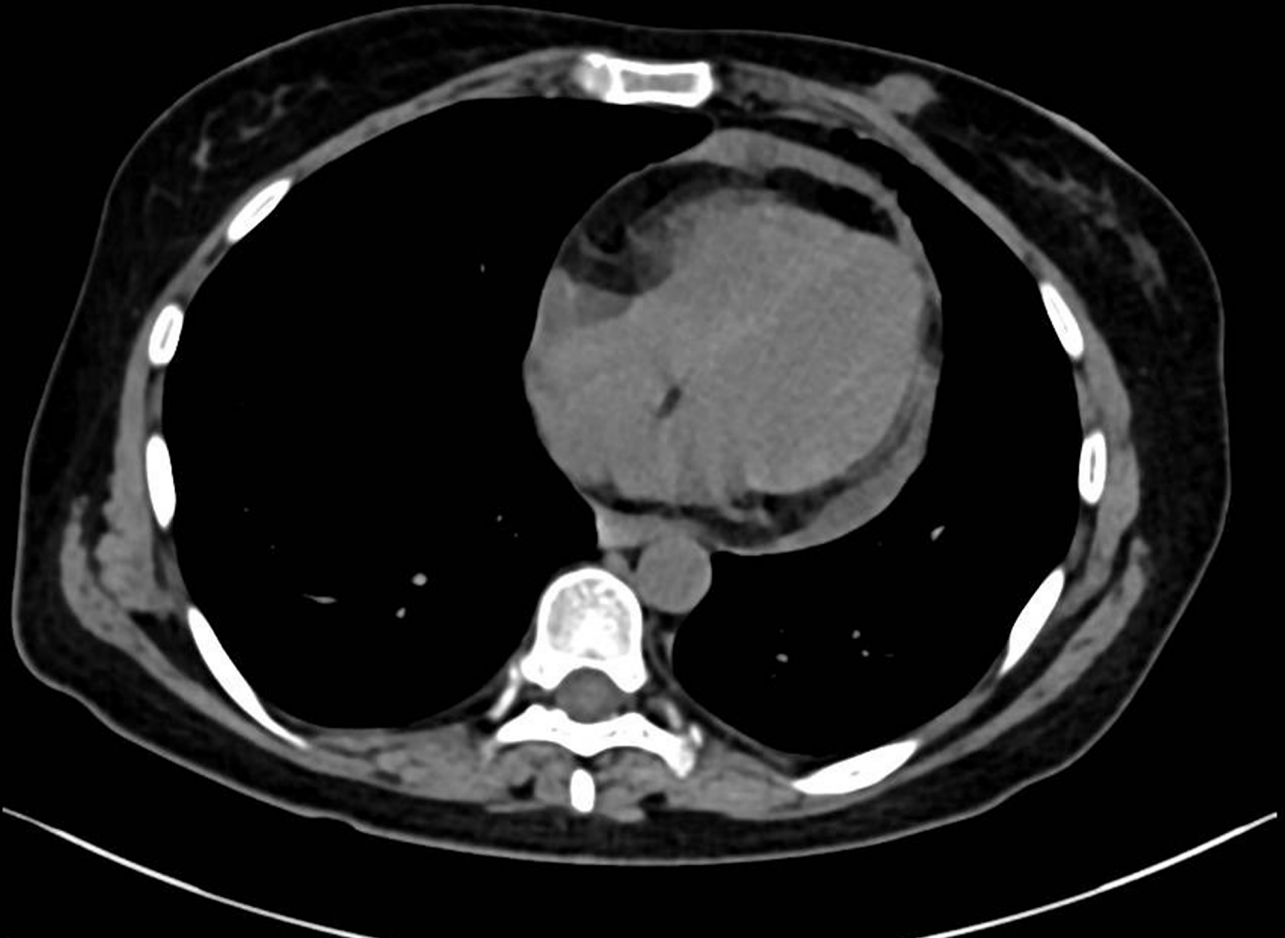
Chest CT after treatment.

The patient started chemotherapy combined with anti-angiogenic therapy treatment (paclitaxel + cisplatin + bevacizumab) after the diagnosis of the mass. After four cycles of therapy, the breast and lung tumors were smaller than before. Considering that the cervical cancer has metastasis to the breast and the left porta of the lung that mean short-term survival, the patient refused to accept surgery. The treatment of IB was accepted because it can deliver in a short period with little complication. Then, under local anesthesia, two and three needles were used in the first and second IB. Two single-fraction implants of 20 Gy with an interfractional interval of 4 days were delivered. We contoured gross tumor volume (GTV) and organ at risk (OAR) and designed the radiotherapy plan in the treatment planning system Oncentra (Elekta AB, Stockholm, Sweden). We used Flexitron HDR for the treatment and iridium-192 as the treatment source. The needles were removed after the treatment was finished per fraction and there were no treatment-related complications. The three-dimensional dose distribution and Dose Volume Histogram (DVH) are shown in **Figures 4**, **5**, and dose parameters are described in **Table 1**. In addition, the left porta of the lung received external beam radiotherapy to a dose of 62.4 Gy in 26 fractions, 2.4 Gy per fraction to left hilar with 6-MV photons. We optimized the VMAT (volume-modulated arc therapy) radiotherapy plan in the treatment planning system, Monaco, and deliver the plan in the Elekta linear accelerator (Elekta AB, Stockholm, Sweden). The three-dimensional dose distribution and DVH are shown in **Figure 6**, and dosimetric parameters are described in **Table 1**. Six months later, the chest CT showed that the enlarged lymph nodes in the left hilar and mediastinum disappeared, and the mass of left breast significantly shrunk. According to the RECIST standard for tumor efficacy evaluation, the left hilar metastasis reached complete remission (CR), and the left breast metastases reached partial remission (PR) (**Figure 7**), but the patient refused to accept to continue treatment.

**Table 1:** Dose-volume histograms of the Planning Target Volume (PTV), lungs, and heart, during external beam radiotherapy of the left porta of the lung, and GTV during the first and second interstitial brachytherapy.

VMAT plan	DVH value	Percentage
**PTV**		
	V_62.4Gy_ (%)	97.6
**Lungs**		
	V_5Gy_ (%)	45.8
	V_10Gy_ (%)	32.6
	V_15Gy_ (%)	21.2
	V_20Gy_ (%)	14.1
**Heart**		
	V_5Gy_ (%)	62.9
	V_20Gy_ (%)	46.6
	V_30Gy_ (%)	35.3
	V_40Gy_ (%)	14.2
**IB plan**	**First fraction of IB**	**Second fraction IB**
**GTV**		
D_90%_ (Gy)	20	20.1
D_100%_ (%)	90	90.3
D_150%_ (%)	68.6	62.3
D_200%_ (%)	50.1	42.2

## Discussion

Cervical cancer is the most common malignant tumor of female reproductive tract. Local invasion and lymph node metastasis are the most common most common spread pattern of cervical cancer. The distant metastasis of cervical cancer usually occurs in the lung, multiple sites, omentum, bone, and liver in a report using PET-CT to evaluate the distant metastasis of initial staging of cervical carcinoma (4). In addition, patients with multiple metastatic sites have a poor overall survival. Metastasis of cervical cancer to the breast is rare. At present, there are only dozens of cases of breast metastasis of cervical cancer (5, 6), and most of them are in the late stage of the disease. We report a case of breast metastasis associated with pulmonary metastasis of early cervical cancer (stage IIB), which is extremely rare in clinic.

As far as the current study is concerned, the occurrence of breast metastasis from cervical cancer is irregular. Different reports gave different time of diagnosis, and the temporal relationship between diagnosis and treatment of cervical carcinoma and detection of breast mass varied from 0 months to more than 9 years. There is no obvious preference for the stage of cervical cancer, even patients with low stage tumors (stages I and II) would metastasize to breast (5). In our report, this case occurred in a patient with stage IIB disease. This patient’s breast mass occurred in the third year of post-treatment follow-up, and the patient was presented with breast mass as the first manifestation, which had a clinical presentation indistinguishable from PBC (5, 7) and had no abnormalities identified on gynecological examination and MRI, which posed difficulties in distinguishing MBC from PBC, and their treatment is quite different, so the diagnosis must first be established.

Histopathological examination is very important for accurate diagnosis that can accurately identify the type of tumor. Squamous cell carcinoma is more than 50% of cervical cancer histology in the reported cases of cervical cancer and breast metastasis (7). However, the report of histopathology is subjective and inaccurate in distinguishing MBC from PBC (8). Immunohistochemistry (IHC) is the key to distinguish primary lesions from metastases. Therefore, the use of IHC staining plays a crucial role in the diagnosis of metastatic tumors (3, 9). For IHC markers identifying breast cancer and determining prognosis, a literature review of metastasis of cervical cancer to breast was made by Mangla et al. (5). Immunohistochemical analysis of primary breast squamous cell carcinomas revealed immunoreactivity for neuron-specific enolase, E-cadherin, thyroid transcription factor-1, Bcl-2, synaptophysin, and chromogranin. Primary squamous cell carcinoma of the breast is characterized by CK-7 positive and CK-20 negative (7, 10). In addition, some histological features are helpful in distinguishing primary and metastatic tumors, such as atypical histologic features of PBC, a well-circumscribed tumor with multiple satellite foci, lack of intraductal component, and numerous lymphatic emboli (11). P16 is associated with high-risk human papilloma virus (HPV), and it is not seen in normal cervical epithelium. P16 combined with Ki-67 is useful in the diagnosis of small high-grade cervical intra-epithelial neoplasia (12). In this patient, IHC staining is strongly positive for p16 and Ki-67, strongly pointing to the possibility of a cervical origin and supporting the notion that the breast squamous cell carcinoma was a metastasis of the cervical squamous cell carcinoma.

In the treatment of metastatic cervical cancer, cisplatin-based chemotherapy combined with radiotherapy is an effective treatment for locally advanced and partially metastatic cervical cancer. For patients with cervical cancer with extensive metastasis, the main goal of chemotherapy is to prolong life and to increase quality of life. As a single agent or combination therapy, cisplatin is the most widely used chemotherapy drug for metastatic cervical cancer, and it can be combined with molecular targeted drugs (13). Because of the rarity of breast metastasis of cervical cancer and the lack of clinical reports, we cannot judge the best treatment method at present. We can only formulate personalized treatment according to the characteristics of patients’ own tumors. Although different treatment modalities were provided in different case reports, the overall survival rate after detection of breast metastasis was always low in all reports (5). In this case, for patient EGFR(+), we decided to use chemotherapy and anti-angiogenic therapy (paclitaxel + cisplatin + bevacizumab) combined with radiotherapy as a systemic treatment. The left hilar metastasis achieved CR, and the left breast metastases achieved PR by RECIST tumor response evaluation criteria after all the treatment. Therefore, we point out that the treatment modality can be decided by the results of IHC; meanwhile, chemotherapy combined with immunotherapy and radiotherapy is a strategy worth borrowing for breast metastasis of cervical cancer.

The breast metastasis mechanism of cervical cancer is still unclear. On the basis of the literature, chemokines play an important role in tumor development. They participate in tumor growth, progression, and metastasis (14). Chemokines recruit specific lymphocyte populations to certain tissues through innate or acquired immune responses to regulate the migration of white blood cells. Cancer cells express appropriate receptors and transfer cancer cells to other locations (15). It has been reported in the literature that the expression of chemokines CXCR4 and CCR7 may be related to tumor metastasis (14). In addition, the extracellular matrix (ECM) also plays an important role in the metastasis and colonization of tumors. ECM is a supramolecular aggregate composed of extracellular proteins, proteoglycans, and glycoproteins. A tumor induces ECM of its primary site and distant organs to remodel, such as tissue stiffness, increased connective tissue proliferation, and basement membrane lesion, which is one of the influencing factors of tumor cell metastasis (16). Metastatic dissemination may have occurred by either a hematogenous or a lymphatic route. Lymphatic metastasis to the breast may occur by several routes and is dependent upon retrograde flow. One route would be *via* the para-aortic lymph nodes to the subclavian lymph node chain with subsequent back flow to the axillary lymph node groups and then to the subareolar and circumareoloar plexuses with final termination within the perilobular and interlobular plexuses within the breast. An alternate route would involve retrograde flow from the anterior parasternal lymph nodes to the medial aspect of the breast (17, 18).

Breast metastasis of cervical cancer is uncommon, whose frequencies was 0.5%–6.6% reported in clinical and autopsy studies (19). Speert and Greeley reported one patient and could find only four cases in the literature (20). Film-screen mammogram combined with ultrasound can narrow the list of differential diagnoses (21). Fine-needle aspiration or excisional biopsy of the lesion and IHC should be performed to confirm the diagnosis (17, 22–26). It has been reported that Cis-platinum chemotherapy and local radiotherapy resulted in complete response in breast metastasis of cervical cancer (19, 27). However, because of too few cases, there is no relevant treatment guideline. In this case, the innovation of this case lies in the treatment plan. We use immunocytochemistry to establish the diagnosis and to summarize the pathway and mechanism of breast metastasis of cervical cancer. At the same time, chemotherapy scheme and IB have achieved good results. The most important point is that IB was proposed as a local treatment for isolated breast metastasis of cervical cancer, which achieved good curative effect in a short treatment period. We hope that our presented case can provide a reference for clinic.

## Conclusions

In summary, breast metastasis of cervical cancer has no special clinical manifestations, and it also lacks specificity in diagnostic imaging. IHC is the gold standard for distinguishing MBC from PBC. For breast cancer based on medical history of malignant tumor, the source of the tumor must be clarified to get correct treatment. Certainly, exploring the mechanism of breast metastasis of cervical cancer will provide more powerful help for diagnosis and treatment. In addition, IB is an effective treatment way for single breast metastasis tumor, which can be completed in a relatively short time and with minimal trauma.

## Data availability statement

The original contributions presented in the study are included in the article/supplementary material. Further inquiries can be directed to the corresponding author.

## Author contributions

ZJ-y and ZG-p conducted the case data collection and the manuscript writing. ZG-p performed the review of literature and revised the manuscript. WX contributed to the planning, data collection. ZS-m contributed to data analysis. ZZ-g critically reviewed the paper and made important contributions to the case interpretation. All authors contributed to the article and approved the submitted version.

## Acknowledgments

We would like to express our gratitude to all those who helped us during the writing of this thesis.

## Conflict of interest

The authors declare that the research was conducted in the absence of any commercial or financial relationships that could be construed as a potential conflict of interest.

## Publisher’s note

All claims expressed in this article are solely those of the authors and do not necessarily represent those of their affiliated organizations, or those of the publisher, the editors and the reviewers. Any product that may be evaluated in this article, or claim that may be made by its manufacturer, is not guaranteed or endorsed by the publisher.
